# Editorial: Data-driven technologies for future healthcare systems

**DOI:** 10.3389/fmedt.2023.1183687

**Published:** 2023-05-24

**Authors:** Md Anisur Rahman, Alireza Moayedikia, Uffe Kock Wiil

**Affiliations:** ^1^ School of Computing, Mathematics and Engineering, Charles Sturt University, Bathurst, NSW, Australia; ^2^ Department of Business Technology and Entrepreneurship, Swinburne University of Technology, Hawthorn, VIC, Australia; ^3^ SDU Health Informatics and Technology, University of Southern Denmark, Odense, Denmark

**Keywords:** data mining, data analytics, machine learning, healthcare systems, health outcome

**Editorial on the Research Topic** Data-driven technologies for future healthcare systems

The use of data in modern healthcare systems is increasing rapidly with the increased availability and volume of digital health data and the advancement of state-of-the-art artificial intelligence (AI) such as machine learning and deep learning techniques. Research involving health data has received a great deal of attention in recent years from the research community. Development and deployment of novel and existing AI techniques can have huge benefits for the healthcare systems including early detection, monitoring, prevention, prediction, diagnosis, treatment, and administration of diseases of patients (1). AI techniques will play an important role in the development of future data-driven tools to help both healthcare professionals and patients take more informed decisions to improve health outcome.

The main goal of this Research Topic was to explore novel and existing data-driven techniques in healthcare relevant for development of innovative data-driven solutions, i.e., to be used for:

• **intelligent monitoring and decision support systems** such as the PATINA decision support tool that based on monitoring of existing municipality care data can help prevent hospitalization of frail older adults living in their own homes (2).
• **predictive models for prevention, early detection, diagnosis, treatment, and prognosis** such as the prognostic tool that based on infrared thermography and deep learning techniques can help identify high-risk patients during triage at the Emergency Department (3).
• **digital health systems offering novel personalized data collection, treatment plans and interventions** such as the eMindYourHeart web-based intervention for depression and anxiety in patients with ischemic heart disease attending cardiac rehabilitation (4).

The common thread in the above three examples is that data-driven technologies should be used to support humans in their current work (i.e., decision support systems) rather than replace humans (i.e., decision making systems). This also implies that the AI-based decision support systems should provide their recommendations in a form that is interpretable and understandable by humans (i.e., explainable AI). Failing to do so limits the usability of and trust in such systems (i.e., black box systems). These aspects are particular important to address in work settings such as healthcare where decisions have impact on human lives. While the opportunities of using AI for health are many, currently the examples of successful use in clinical practice are still limited due to various challenges as documented in detail in two recent review papers (5, 6).

Papers within this Research Topic focus on challenges, limitations, opportunities, and potential impact for future healthcare systems from practical, experimental, or theoretical perspectives. Papers emphasize how the stakeholders (i.e., patients and their relatives, healthcare professionals, hospital administrators, etc.) benefit from the proposed data-driven solutions.

We received 13 submissions from a variety of countries including United States, China, Republic of Korea, Pakistan, Australia, Kazakhstan, Iran, and Czechia. Six quality papers were accepted. We provide a brief introduction to each accepted paper below.

Levy et al. used 12 machine learning algorithms to predict hospital acquired pressure injuries (HAPI). The data for the paper was based on a retrospective study. The results show that a logistic regression algorithm performs better than the other used classification algorithms to predict HAPI.

Lyu et al. presented a model to predict patient choice in medical decision-making. Three classifications algorithms were used in their study. The research aims to promote the development and application of a patient-centric medical decision-making system to obtain better patient outcome.

Li et al. developed an artificial intelligence tool for diagnosis and treatment of ankylosing spondylitis. It is shown that the performance of the tool surpassed that of human experts, and that the model also significantly improved the experts' diagnostic accuracy.

Kim et al. used machine learning models to automate the classification of sleep stages and estimation of sleep efficiency based on heart rate variability derived features and acceleration features processed from actigraphy data.

Huang et al. developed and validated predictive machine learning models for risk stratification for 28-day in-hospital mortality of elderly patients with ischemic stroke who were admitted to the intensive care unit.

Liu et al. used natural language processing and machine learning algorithms to develop an intelligent medical guidance and recommendation model based on online patient-physician communication data.

In summary, the six accepted papers demonstrate how data-driven technologies can be used to assist healthcare professionals and patients to better manage their disease by providing novel decision support. Showing the potential is only the first step in successful clinical use of AI-based techniques. However, we sincerely hope to see an increased focus in future work to bring the promising solutions into real clinical practice to fully unlock the potential to improve health outcomes in the future.
